# Template-Free Synthesis of One-Dimensional g-C_3_N_4_ Chain Nanostructures for Efficient Photocatalytic Hydrogen Evolution

**DOI:** 10.3389/fchem.2021.652762

**Published:** 2021-03-15

**Authors:** Mingyi Zhang, Ye Sun, Xin Chang, Peng Zhang

**Affiliations:** ^1^ Key Laboratory for Photonic and Electronic Bandgap Materials, Ministry of Education, School of Physics and Electronic Engineering, Harbin Normal University, Harbin, China; ^2^ School of Materials Science and Engineering, Zhengzhou University, Zhengzhou, China

**Keywords:** g-C3N4, one-dimensional (1D), photocatalytic, template-free, hydrogen evolution

## Abstract

The development of graphite-carbon nitride (g-C_3_N_4_) photocatalyst is of great significance for various visible utilization applications. Control the nanostructures of g-C_3_N_4_ can tailor its photocatalytic performance. In this paper, one-dimensional chain-like g-C_3_N_4_ was successfully synthesized by heat-induced polymerization of melamine which was saturated in ethylene glycol. The photocatalytic hydrogen production rate (HER) of the prepared g-C_3_N_4_ chain enhanced about 3 times than that of bulk g-C_3_N_4_, increasing from 9.6 μmolh^−1^ to 28.7 μmolh^−1^. The improved photocatalytic activity of the g-C_3_N_4_ chain was attributed to the advantages of porosity and nanostructure. The extraordinary nanopores result in an enlarged specific surface area for adsorption and the production of abundantly available channels for charge transfer. The one-dimensional chain-like structure can facilitate the exposure of internal/external active sites as many as possible, and induce the directional migration of charge carriers.

## Introduction

With the rapid pace of industrialization and urbanization growing in the past few decades, global crises related to environmental degradation and energy shortage have become the most critical topics to the world and threaten the survival environment of humankind (Yu et al., 2017; Yu et al., 2019). Photocatalytic water splitting for hydrogen evolution has been considered as a sustainable strategy to convert and store plentiful solar energy for future energy requirements (Wang et al., 2009; Wang et al., 2012; Ong et al., 2016). In the last several decades, graphitic carbon nitride (g-C_3_N_4_) the organic semiconductor showed a specific graphite-like sp^2^-bonded C–N structure that exhibits significant potential in the field of CO_2_ conversion, water splitting, and environmental remediation on account of its inexpensive preparation, brilliant visible light response, thermal stability, and well-developed electronic band structure (Ding et al., 2018; He et al., 2019; Rong et al., 2020; Shu et al., 2020; Zhang D. et al., 2020). However, g-C_3_N_4_ accompanied by the defaults such as poor quantum efficiency, low specific surface area, and rapid charge recombination have adversely affected the photocatalytic application of g-C_3_N_4_.

A large number of strategies acting to resolve these problems and reinforce the photocatalytic performances of g-C_3_N_4_, involving non-metal element doping [S (Cao et al., 2018), O (Jiang et al., 2019), V (Ding et al., 2013), B (Yan et al., 2010), etc.], noble metals decoration [Pd (Wang et al., 2011) Pt (Maeda et al., 2009) and Au (Li et al., 2012)], heterojunction designing (Liang et al., 2019; Shi et al., 2020a; Shi et al., 2020b) and coupling with graphene (Li et al., 2013; Han et al., 2017). Expect for the capacity of g-C_3_N_4_ to collaborate with other materials, layers structure configuration is deemed as a prospective method to compound g-C_3_N_4_ as they can accelerate the diffusion of reactant, strengthen the light harvesting, expand the exposed surface areas, and promote the charge delivery.

Generally speaking, microstructural g-C_3_N_4_ photocatalysts with controllable morphology and structure can be divided into six categories: mesoporous (Zhao et al., 2018; Chen et al., 2019), nanosheets (Yang et al., 2017; Gao et al., 2018), nanorods (Cui et al., 2012; Bai et al., 2013), nanotubes (Gao et al., 2012), and nanospheres (Zheng et al., 2015). Among them, one-dimensional (1D) nanostructured photocatalyst, especially fabricating 1D photocatalyst with large surface area, is especially interested as the charges can vectorially transfer along with the 1D structure (Tahir et al., 2014).

After discovering the carbon nanotubes, 1D nanostructures (lines, rods, tubes, strips, fibers, etc) have attracted extensive attention from researchers. 1D nanostructures have greatly satisfied the increasing demand for microelectronics and optoelectronic devices such as optical waveguides, field-effect transistors, and photodetectors in recent years. 1D nanostructures showed brilliant phonon, gas sensitivity, field emission, photoconductivity, and electron transport performance due to their higher surface volume ratio and more active position. Furthermore, the growth of 1D nanostructures has an immense effect on improving the mechanical energy, thermal and electrical capabilities of materials. Because nanoparticles in 1D nanostructures are interconnected in three dimensions, an extremely fast interparticle, vectorially transport of photogenerated charge carriers (electrons and holes) is likely to emerge through the grain boundaries. This represents that the redox reaction sites are far away related to the photoexcitation sites, which seems to be responsible for the high activities of photocurrent generation and hydrogen production.

In the present work, 1D g-C_3_N_4_ chain nanostructures were obtained by regulating the saturation of melamine and ethylene glycol solution for photocatalytic hydrogen evolution. The enhanced photocatalytic properties are known to be caused by an extension of the life of the photoinduced charge carrier. In addition, increasing specific surface area is also a vital factor in promising photocatalytic performance. Owing to the composite method is convenient, environmentally friendly, and low-cost, it is suitable for an expanded range of practical applications.

## Experimental

### Synthesis of the g-C_3_N_4_ Chain Nanostructures

The one-dimensional g-C_3_N_4_ chain nanostructures were prepared by a controllable approach. Firstly, excessive amounts of melamine powders (3.0 g) were dissolved in 60 ml of ethylene glycol to form a saturated solution at room temperature. Subsequently, add 1 ml of concentrated nitric acid solution to 59 ml of water, drop by drop add the solution to 20 ml of supernatant and stir continuously until a white flocculent precipitate is obtained. The white flocculent precipitate was bleached with ethanol five times to remove nitric acid and ethylene glycol. Lastly, the collected sample was transferred into the muffle furnace for heating 2 h at 550°C with a heating rate of 20°C·min^−1^. Meanwhile, as the contrast sample, the bulk g-C_3_N_4_ was obtained by the heating process at 550°C for 2 h with a heating rate of 20°C·min^−1^ of the melamine powders.

### Characterization

The crystal structure was characterized by X-ray diffraction (XRD, D/max2600, Rigaku, Japan) using the Cu Ka radiation (k = 1.5418 Å). The morphologies of one-dimensional g-C_3_N_4_ chain nanostructures were characterized by scan electron microscopy (SEM, SU70, Hitachi, Japan). And specific surface areas of the one-dimensional g-C_3_N_4_ chain nanostructures were measured by a Micromeritics ASAP 2010 instrument and analyzed by the Brunauer–Emmett–Teller (BET) method. Photoluminescence (PL) spectra of photocatalysts were performed on a Jobin Yvon HR800 micro-Raman spectrometer including a 325 nm line from a He-Cd laser. UV-Vis diffuse reflectance spectra (DRS) of the samples are obtained by using a UV-Vis-IR spectrometer (Perkin-Elmer, Lambda 850). Photocurrent measurements were characterized by CHI 660 E electrochemical workstation (Chenhua, Shanghai) by applying a three-electrode cell accompanied by a visible light source. A platinum filament, Ag/AgCl electrode, and 0.2 M of Na_2_SO_4_ were acted as the counter electrode, reference electrode, and an electrolyte solution, separately. The as-fabricated sample mixed a certain amount of Nafion solution was painted on FTO glass as a working electrode (the effective area was 1 cm × 1 cm).

### Photocatalytic Test

Photocatalytic H_2_ evolution was injected into a 250 ml of quartz reactor with a visible-light source irradiation. As a typical synthesis experiment, 0.1 g of photocatalyst with a certain Pt cocatalyst (1 wt%) was dispersed in a mixed solution of aqueous solution (90 ml) and methanol (10 ml). The amount of H_2_ evolution was measured in a gas chromatograph (GC-2014C Shimadzu Corp., N_2_ as carrier gas). The time interval of sampling was performed at 40 min during the water splitting process.

## Results and Discussion

**FIGURE 1:**
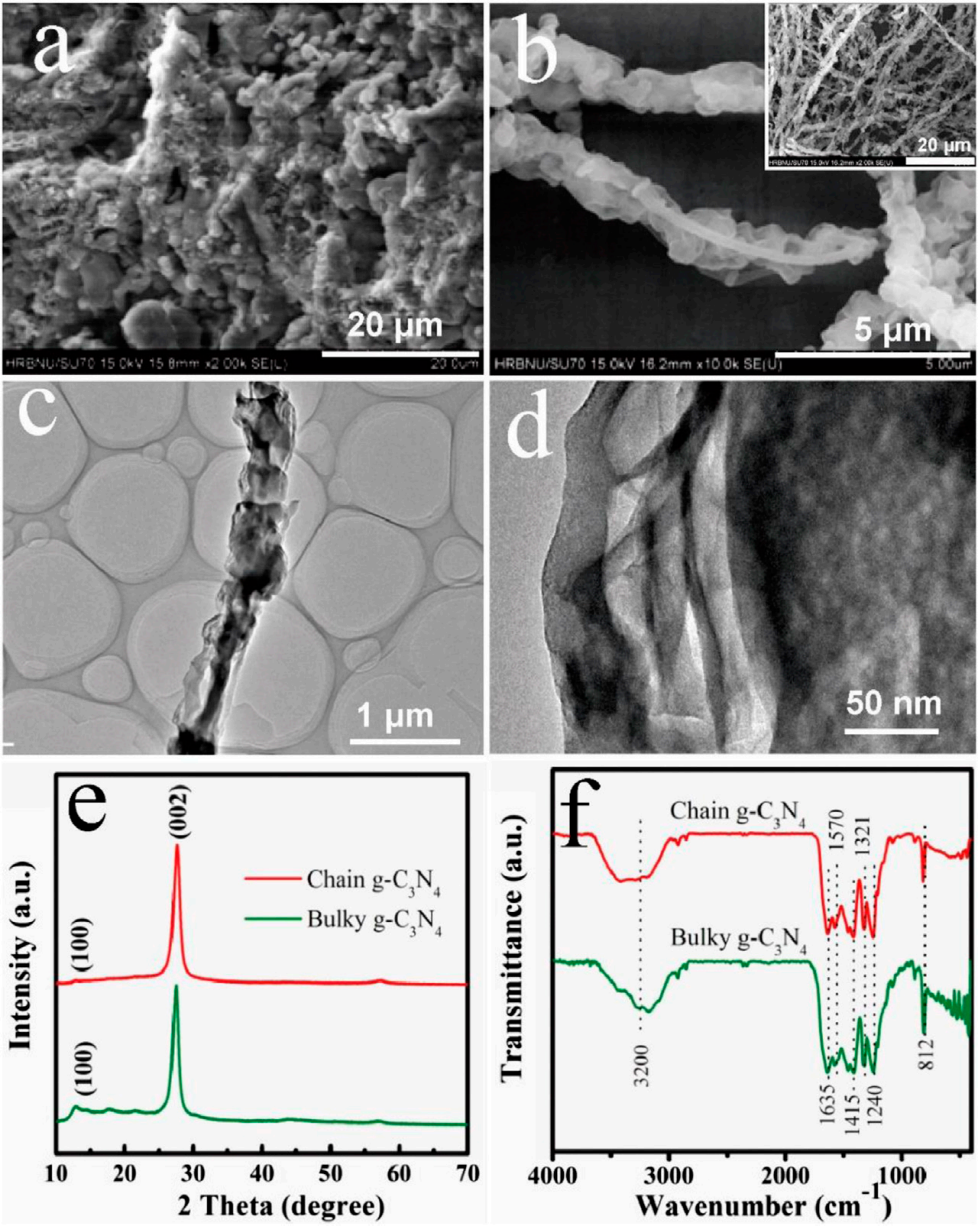
SEM images of **(A)** bulky g-C_3_N_4_ and **(B)** chain g-C_3_N_4_ at low and high magnification, and **(C, D)** TEM image of chain g-C_3_N_4_ at low and high magnification; **(E)** XRD and **(F)** FT-IR patterns of bulky g-C_3_N_4_ and pearl-chain g-C_3_N_4_.

The morphology of the as-fabricated products was scrutinized by scanning electron microscope (SEM). Figure 1A depicts the SEM image of the as-prepared bulk g-C_3_N_4_, in which the sample possesses a hierarchical component with a particle size of greater than 20 μm. However, in Figure 1B, we found that the morphology of g-C_3_N_4_ changed greatly compared with the bulk structure, and a new one-dimensional chain structure appeared. It can be illustrated from Figure 1B that the lengths of these irregular-oriented chains g-C_3_N_4_ one-dimensional structure could reach dozens of micrometers, and the diameters of those fibers range from 1 to 2 µm. Each one-dimensional structure was separated from each other, and further secondary structures could be found. Through the observation of the morphology characteristics of the two structures, we can infer that the chain g-C_3_N_4_ material will possess a vast specific surface area, which is more conducive to enhance the photocatalytic performance. The transmission electron micrographs of chain g-C_3_N_4_ are shown in Figures 1C,D. We can see the chain g-C_3_N_4_ presents a similar sponge hole that exists on the surface of the chain g-C_3_N_4_, the morphology can effectively improve the capacity in the course of the photocatalytic reaction area. Meanwhile, the product can shorten the time and distance of the charge transfer, and promote the charge separation order to improve the photocatalytic activity of the material. As we all known, in the pyrolysis period of supramolecular precursor, the by-products were formed with varieties of gases gradually released, giving rise to the nitrogen defects obtained in the framework of g-C_3_N_4_.

X-ray diffraction (XRD) patterns for chain g-C_3_N_4_ and bulky g-C_3_N_4_ are expressed in Figure 1E. Obviously, both patterns contain two diffraction peaks, which are located at 13.2° and 27.6°, respectively. The former peak at 13.2° could be indexed as (100) lattice plane, which is associated with interlayer stacking. The corresponding interlayer spacing value has been calculated to be 0.676 nm. And the later peak at 27.6° is a feature interlayer stacking peak of aromatic systems, which could be indexed as (002) lattice plane. The calculated interplanar distance of aromatic units is 0.326 nm (Li et al., 2020a; Zhang et al., 2020b).

Figure 1F shows the Fourier transform infrared (FT-IR) spectra of the as-prepared samples. The FT-IR spectra of the synthesized chain g-C_3_N_4_ were compared to the bulky g-C_3_N_4_ exhibit with similar characteristics. The FT-IR the bands located at 1,240, 1,321, 1,415 cm^−1^, and 1,570 cm^−1^ are mainly from the typical stretching modes of C-N heterocycles. And the band at 810 cm^−1^ is attributed to out-of-plane bending modes of C-N heterocycles. The C-N stretching mode has IR band at 1,635 cm^−1^. And the broadband near 3,200 cm^−1^ corresponds to the stretching modes of terminal NH_2_ or NH groups at the defect sites of the aromatic ring (Li et al., 2020b; Li et al., 2020c).

**FIGURE 2:**
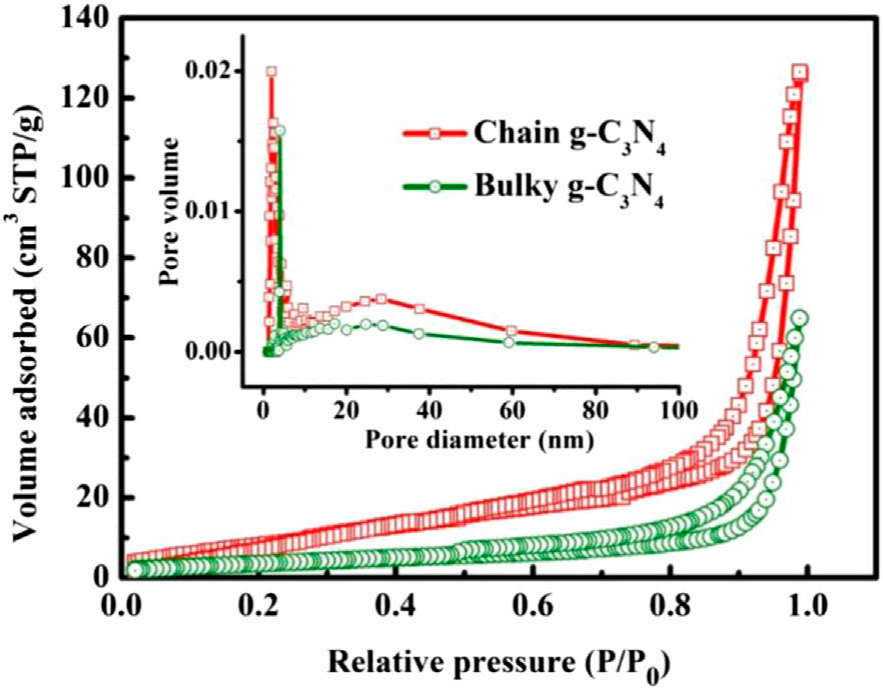
BET nitrogen adsorption/desorption isotherms of bulky g-C_3_N_4_ and chain g-C_3_N_4_.

The exposed surface area and pores distribution of chain g-C_3_N_4_ and bulky g-C_3_N_4_ were further researched. The adsorption-desorption isotherm curve of chain g-C_3_N_4_ and bulky g-C_3_N_4_ demonstrate the type IV curve, as shown in Figure 2, indicating the presence of uniform mesoporous with high specific surface area and large total pore volume. The specific surface area of chain g-C_3_N_4_ was counted to be 47.85 m^2^g^−1^ through the Brunauer-Emmett-Teller (BET), which is approximately three times larger than pure bulky g-C_3_N_4_ (16.15 m^2^g^−1^). The inset in Figure 2 exhibits the pore-size distribution of the chain g-C_3_N_4_ and bulky g-C_3_N_4_. The pore distribution for chain g-C_3_N_4_ is mainly located at 2.46 nm, and the pore volume is 0.233 cm^3^g^−1^. But for the bulky g-C_3_N_4_, the pore volume is just 0.097 cm^3^g^−1^. The mesoporous structure and large surface area of the chain g-C_3_N_4_ are conducive to the absorption of more active substances and reactants on the surface, enhancing the photocatalytic reaction. Therefore, we can assume that the chain g-C_3_N_4_ material can have superior photocatalytic activity.

**FIGURE 3:**
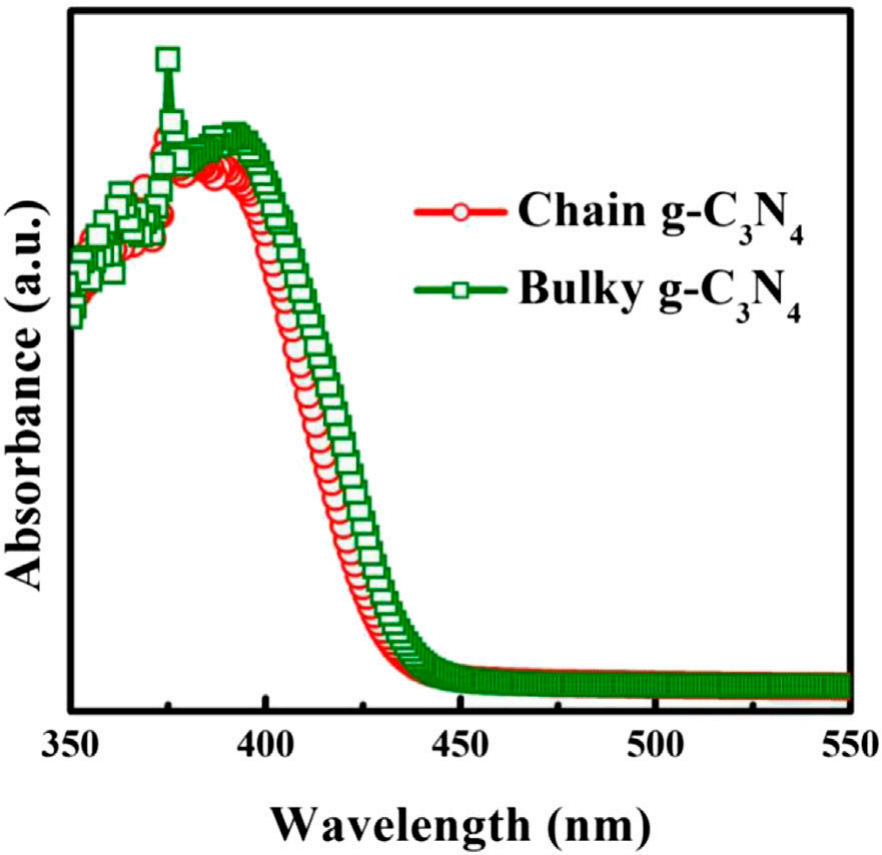
UV-vis absorption spectra of bulky g-C_3_N_4_ and chain g-C_3_N_4_.

The UV-vis diffuse reflectance spectroscopy illustrated by Figure 3. from the (αhv)1/2 vs. photon energy (hv) plot (Hou et al., 2020; Li et al., 2020d; Wang et al., 2020a; Zhang et al., 2020a; Zhang Y. et al., 2020), the optical bandgap of chain g-C_3_N_4_ and bulk g-C_3_N_4_ was calculated to be approximate 2.88 and 2.93 eV, respectively. When the particle size descends to a certain value, the electron energy level near the Fermi energy level of the metal changes from quasi-continuous to discrete energy level (Miao et al., 2019; Huang et al., 2020b; Wu et al., 2020; Zhang S. et al., 2020), and the highest occupied molecular orbital (HOMO) and lowest unoccupied molecular orbital energy level (LUMO) of the nano-semiconductor particles have discontinuous energy gap, which leads to the blue shift of the chain g-C_3_N_4_.

According to mentioned above, the prepared chain g-C_3_N_4_ has a one-dimensional superstructure with a wide surface area, making it a more suitable candidate material for photocatalytic H_2_ production. To evaluate the photocatalytic performance of chain g-C_3_N_4_ photocatalyst with the visible light irradiation, the H_2_ production performance was tested and compared with that of bulky g-C_3_N_4_ photocatalytic performance was compared.

**FIGURE 4:**
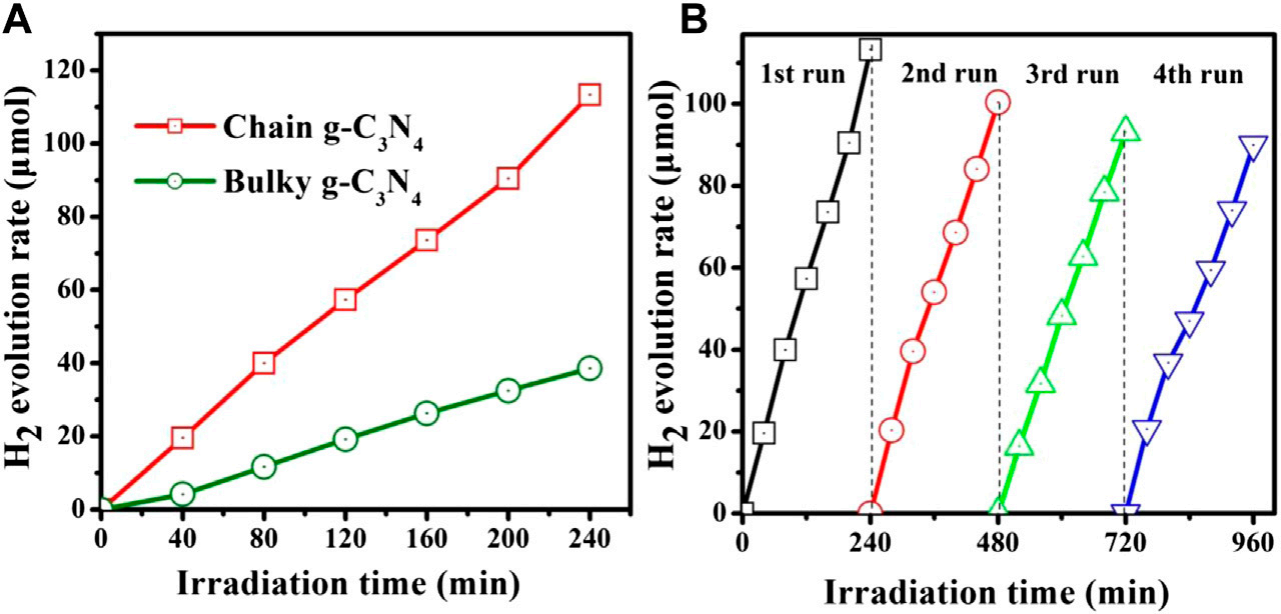
**(A)** The amount of hydrogen evolution over the bulky g-C_3_N_4_ and chain g-C_3_N_4_, and **(B)** cycling test of photocatalytic hydrogen evolution under visible light.

As the result shown in Figure 4A, the H_2_ evolution rate of the bare bulky g-C_3_N_4_ sample was measured to be 9.6 μmolh^−1^, separately. However, the photocatalytic properties of chain g-C_3_N_4_ were markedly improved, and the H_2_ generation rate increased as high as 28.7 μmolh^−1^. The chain g-C_3_N_4_ sample shows rather superior photocatalytic activity for H_2_ evolution, which could be ascribed to its vaster surface area (Figure 2). Figure 4B shows the stability of the photocatalytic H_2_ production system using chain g-C_3_N_4_ as the photocatalyst under visible light irradiation. Therefore, we studied the chemical stability of chain g-C_3_N_4_, as described in Figure 4B. After four consecutive cycle experiments, we found that the evolution rate of the chain g-C_3_N_4_ did not decline significantly, indicating that the material has good chemical stability.

**FIGURE 5:**
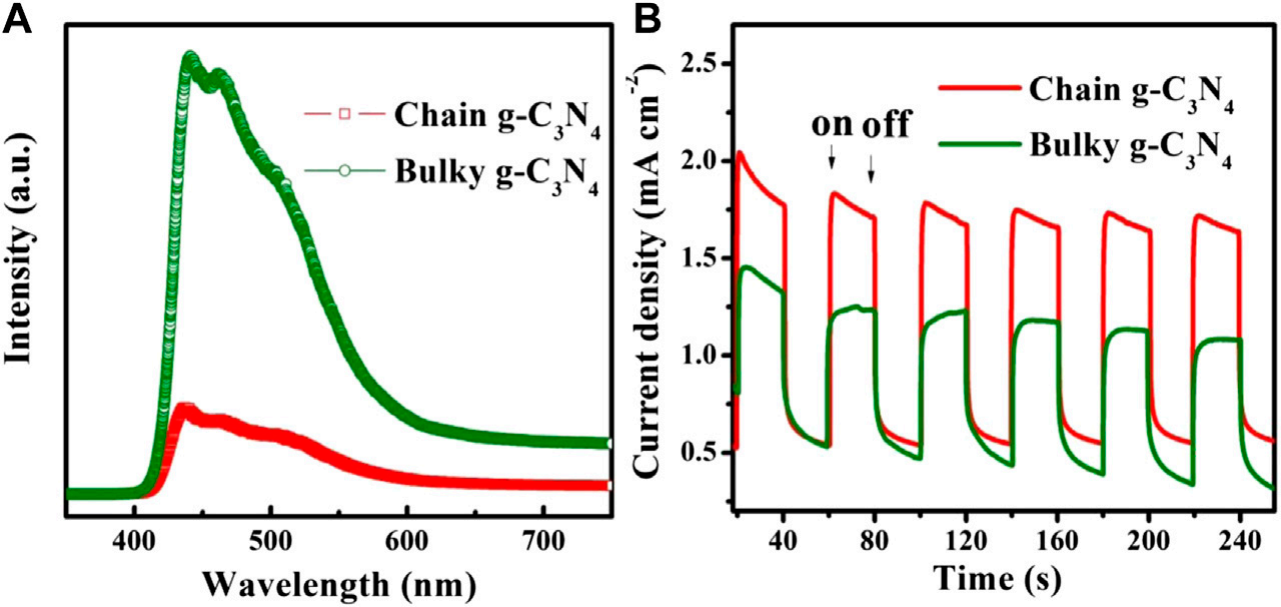
**(A)** PL spectra and **(B)** photocurrents spectra of bulky g-C_3_N_4_ and chain g-C_3_N_4_.

The key factor of photocatalytic reaction is the effective generation and rapid separation of photoexcited carriers (Huang et al., 2020a; Li X. et al., 2020; Wang L. et al., 2020; Wang et al., 2020b; Zhang et al., 2020c). The optical performances of the samples were measured by photoluminescence (PL). The intensity of PL spectra can state the extent of the recombination of photo-generated charges. In Figure 5A, it illustrates bulky g-C_3_N_4_ emerge severe charge recombination, while the PL spectrum of chain g-C_3_N_4_ is intense quenched (Figure 5A). The reorganizing of the photo-generated e-h pairs of chain g-C_3_N_4_ can be restrained. The restraining of the e-h pair recombination is powerfully verified by the increased photocurrents for chain g-C_3_N_4_, as shown in Figure 5B. Apparently, the chain g-C_3_N_4_ was provided with a better separation efficiency of e-h pairs.

To further confirm the ability of several samples to separate and transfer charges and to respond to light (Cheng et al., 2019; Li et al., 2021), we performed photocurrent tests on them, as shown in Figure 5B six cycles were tested under λ > 420 nm xenon lamp. As can be seen from the diagram, two working electrode transient photocurrent response of bulky g-C_3_N_4_ and chain g-C_3_N_4_ were obtained through these six cycles. It can be seen that the transient photocurrent spectrum of the chain g-C_3_N_4_ electrode was much better than the bulky g-C_3_N_4_. Test results indicate that chain g-C_3_N_4_ greatly improves the separation of interface carriers. The photocurrent research consequences coincide with the impedance and photoluminescence research consequences, demonstrating that the chain g-C_3_N_4_ material will have better photocatalytic performance.

## Conclusion

In summary, we proposed an annealing method to achieve bulky g-C_3_N_4_ and chain g-C_3_N_4_ utilizing melamine as reactant materials. The chain g-C_3_N_4_ nanostructures illustrated improvement on the photocatalytic H_2_ production under visible light irradiation owing to unique inimitable one-dimensional structure, high specific surface area, excellent light-harvesting properties, and low recombination rate of electron-hole pairs. This simple preparation and facile composition of one-dimensional g-C_3_N_4_ nanostructures demonstrate a promising candidate for exploring more actual applications of carbon nitride.

## Data Availability

The original contributions presented in the study are included in the article/Supplementary Material, further inquiries can be directed to the corresponding author.

## References

[B28] Bai X. Wang L. Zong R. Zhu Y. (2013). Photocatalytic activity enhanced via g-C3N4 nanoplates to nanorods. J. Phys. Chem. C. 117, 9952–9961. 10.1021/jp402062d

[B11] Cao S. Fan B. Feng Y. Chen H. Jiang F. Wang X. (2018). Sulfur-doped g-C3N4 nanosheets with carbon vacancies: general synthesis and improved activity for simulated solar-light photocatalytic nitrogen fixation. Chem. Eng. Technol. 353, 147–156. 10.1016/j.cej.2018.07.116

[B23] Chen X. Shi R. Chen Q. Zhang Z. Jiang W. Zhu Y. (2019). Three-dimensional porous g-C3N4 for highly efficient photocatalytic overall water splitting. Nano Energy 59, 644–650. 10.1016/j.nanoen.2019.03.010

[B51] Cheng J. Hu Z. Li Q. Li X. Fang S. Wu X. (2019). Fabrication of high photoreactive carbon nitride nanosheets by polymerization of amidinourea for hydrogen production. Appl. Catal. B-Environ. 245, 197–206. 10.1016/j.apcatb.2018.12.044

[B27] Cui Y. Ding Z. Fu X. Wang X. (2012). Construction of conjugated carbon nitride nanoarchitectures in solution at low temperatures for photoredox catalysis. Angew. Chem. Int. Ed. Engl. 51, 11814–11818. 10.1002/anie.201206534 23081850

[B13] Ding G. Wang W. Jiang T. Han B. Fan H. Yang G. (2013). Highly selective synthesis of phenol from benzene over a vanadium-doped graphitic carbon nitride catalyst. ChemCatChem 5, 192–200. 10.1002/cctc.201200502

[B10] Ding J. Xu W. Wan H. Yuan D. Chen C. Wang L. (2018). Nitrogen vacancy engineered graphitic C3N4-based polymers for photocatalytic oxidation of aromatic alcohols to aldehydes. Appl. Catal. B-Environ. 221, 626–634. 10.1016/j.apcatb.2017.09.048

[B26] Gao M. Feng J. Zhang Z. Gu M. Wang J. Zeng W. (2018). Wrinkled ultrathin graphitic g-C_3_N_4_ nanosheets for photocatalytic degradation of prganic wastewater. ACS Appl. Nano Mater. 1, 6733–6741. 10.1021/acsanm.8b01528

[B29] Gao J. Zhou Y. Li Z. Yan S. Wang N. Zou Z. (2012). High-yield synthesis of millimetre-long, semiconducting carbon nitride nanotubes with intense photoluminescence emission and reproducible photoconductivity. Nanoscale 4, 3687–3692. 10.1039/c2nr30777d 22595859

[B21] Han Q. Cheng Z. Gao J. Zhao Y. Zhang Z. Dai L. (2017). Mesh-on-mesh graphitic-C_3_N_4_@graphene for highly efficient hydrogen evolution. Adv. Funct. Mater. 27, 1606352. 10.1002/adfm.201606352

[B9] He S. Xiao K. Chen X.-Z. Li T. Ouyang T. Wang Z. (2019). Enhanced photoelectrocatalytic activity of direct Z-scheme porous amorphous carbon nitride/manganese dioxide nanorod arrays. J. Colloid Interface Sci. 557, 644–654. 10.1016/j.jcis.2019.09.035 31561081

[B40] Hou R. Zhang S. Zhang P. Zhang Y. Zhang X. Li N. (2020). Ti3C2 MXene as an “energy band bridge” to regulate the heterointerface mass transfer and electron reversible exchange process for Li-S batteries. J. Mater. Chem. A 8, 25255. 10.1039/d0ta06695h

[B46] Huang J. Du J. Du H. Xu G. Yuan Y. (2020a). Control of nitrogen vacancy in g-C3N4 by heat treatment in an ammonia atmosphere for enhanced photocatalytic hydrogen generation. Acta. Phys. Chim. Sin. 36, 1905056. 10.3866/pku.whxb201905056

[B43] Huang J. Liu T. Wang R. Zhang M. Wang L. She H. (2020b). Facile loading of cobalt oxide on bismuth vanadate: proved construction of p-n junction for efficient photoelectrochemical water oxidation. J. Colloid Interface Sci. 570, 89–98. 10.1016/j.jcis.2020.02.109 32142906

[B12] Jiang Y. Sun Z. Tang C. Zhou Y. Zeng L. Huang L. (2019). Enhancement of photocatalytic hydrogen evolution activity of porous oxygen doped g-C3N4 with nitrogen defects induced by changing electron transition. Appl. Catal. B-Environ. 240, 30–38. 10.1016/j.apcatb.2018.08.059

[B17] Li X.-H. Wang X. Antonietti M. (2012). Mesoporous g-C_3_N_4_ nanorods as multifunctional supports of ultrafine metal nanoparticles: hydrogen generation from water and reduction of nitrophenol with tandem catalysis in one step. Chem. Sci. 3, 2170–2174. 10.1039/c2sc20289a

[B22] Li Y. Zhang H. Liu P. Wang D. Li Y. Zhao H. (2013). Cross-linked g-C3 N4 /rGO nanocomposites with tunable band structure and enhanced visible light photocatalytic activity. Small 9, 3336–44. 10.1002/smll.201203135 23630157

[B47] Li X. Wang B. Wang B. Yin W. Di J. Xia J. (2020). Cu2+ modified g-C_3_N_4_ photocatalysts for visible light photocatalytic properties. Acta Phys. Chim. Sin. 36, 1902001. 10.3866/pku.whxb201902001

[B32] Li Y. Gu M. Shi T. Cui W. Zhang X. Dong F. (2020a). Carbon vacancy in C_3_N_4_ nanotube: electronic structure, photocatalysis mechanism and highly enhanced activity. Appl. Catal. B-Environ. 262, 118281. 10.1016/j.apcatb.2019.118281

[B34] Li Y. Gu M. Zhang M. Zhang X. Lv K. Liu Y. (2020b). C_3_N_4_ with engineered three coordinated (N3_C_) nitrogen vacancy boosts the production of ^1^O_2_ for Efficient and stable NO photo-oxidation. Chem. Eng. J. 389, 124421. 10.1016/j.cej.2020.124421

[B35] Li Y. Gu M. Zhang X. Fan J. Lv K. Carabineiro S. A. C. (2020c). 2D g-C_3_N_4_ for advancement of photo-generated carrier dynamics: status and challenges. Mater. Today 41, 270–303. 10.1016/j.mattod.2020.09.004

[B38] Li Y. Zhang P. Wan D. Xue C. Zhao J. Shao G. (2020d). Direct evidence of 2D/1D heterojunction enhancement on photocatalytic activity through assembling MoS2 nanosheets onto super-long TiO2 nanofibers. Appl. Surf. Sci. 504, 144361. 10.1016/j.apsusc.2019.144361

[B50] Li K. N. Zhang M. X. Ou X. Y. Li R. N. Li Q. Fan J. J. (2021). Strategies for the fabrication of 2D carbon nitride nanosheets. Acta. Phys. Chim. Sin. 37, 2008010. 10.3866/PKU.WHXB202008010

[B18] Liang S. Zhang D. Pu X. Yao X. Han R. Yin J. Ren X. (2019). A novel Ag2O/g-C_3_N_4_ p-n heterojunction photocatalysts with enhanced visible and near-infrared light activity. Sep. Purif. Technol. 210, 786–797. 10.1016/j.seppur.2018.09.008

[B16] Maeda K. Wang X. Nishihara Y. Lu D. Antonietti M. Domen K. (2009). Photocatalytic activities of graphitic carbon nitride powder for water reduction and oxidation under visible light. J. Phys. Chem. C. 113, 4940–4947. 10.1021/jp809119m

[B44] Miao F. Lu N. Zhang P. Zhang Z. Shao G. (2019). Multidimension‐controllable synthesis of ant nest‐structural electrode materials with unique 3D hierarchical porous features toward electrochemical applications. Adv. Funct. Mater. 29, 1808994. 10.1002/adfm.201808994

[B5] Ong W.-J. Tan L.-L. Ng Y. H. Yong S.-T. Chai S.-P. (2016). Graphitic carbon nitride (g-C_3_N_4_)-based photocatalysts for artificial photosynthesis and environmental remediation: are we a step closer to achieving sustainability? Chem. Rev. 116, 7159–7329. 10.1021/acs.chemrev.6b00075 27199146

[B6] Rong X. Liu S. Xie M. Liu Z. Wu Z. Zhou X. (2020). N2 photofixation by Z-scheme single-layer g-C3N4/ZnFe2O4 for cleaner ammonia production. Mater. Res. Bull. 127, 110853. 10.1016/j.materresbull.2020.110853

[B19] Shi W. Li M. Huang X. Ren H. Yan C. Guo F. (2020a). Facile synthesis of 2D/2D Co3(PO4)2/g-C3N4 heterojunction for highly photocatalytic overall water splitting under visible light. Chem. Eng. J. 382, 122960. 10.1016/j.cej.2019.122960

[B20] Shi W. Liu C. Li M. Lin X. Guo F. Shi J. (2020b). Fabrication of ternary Ag3PO4/Co3(PO4)2/g-C3N4 heterostructure with following Type II and Z-scheme dual pathways for enhanced visible-light photocatalytic activity. J. Hazard. Mater. 389, 121907. 10.1016/j.jhazmat.2019.121907 31879109

[B7] Shu K. Chen F. Shi W. Guo F. Tang Y. Ren H. (2020). Construction of DyVO4/nitrogen deficient g-C3N4 composite for enhanced visible-light photocatalytic activity for tetracycline degradation. Mater. Res. Bull. 124, 110766. 10.1016/j.materresbull.2020.110766

[B31] Tahir M. Cao C. Mahmood N. Butt F. K. Mahmood A. Idrees F. (2014). Multifunctional g-C3N4 Nanofibers: a template-free fabrication and enhanced optical, electrochemical, and photocatalyst properties. ACS Appl. Mater. Interfaces 6, 1258–1265. 10.1021/am405076b 24354285

[B3] Wang X. Maeda K. Thomas A. Takanabe K. Xin G. Carlsson J. M. (2009). A metal-free polymeric photocatalyst for hydrogen production from water under visible light. Nature Mater. 8, 76–80. 10.1038/nmat2317 18997776

[B15] Wang Y. Yao J. Li H. Su D. Antonietti M. (2011). Highly selective hydrogenation of phenol and derivatives over a Pd@carbon nitride catalyst in aqueous media. J. Am. Chem. Soc. 133, 2362–2365. 10.1021/ja109856y 21294506

[B4] Wang Y. Wang X. Antonietti M. (2012). Polymeric graphitic carbon nitride as a heterogeneous organocatalyst: from photochemistry to multipurpose catalysis to sustainable chemistry. Angew. Chem. Int. Ed. Engl. 51, 68–89. 10.1002/anie.201101182 22109976

[B45] Wang L. Zhu C. Yin L. Huang W. (2020). Construction of Pt-M (M = Co, Ni, Fe)/g-C_3_N_4_ composites for highly efficient photocatalytic H_2_ generation. Acta Phys. Chim. Sin. 36, 1907001. 10.3866/pku.whxb201907001

[B36] Wang Y. Liu J. Wang Y. Zhang M. (2020a). CO2 photoreduction to CO/CH4 over Bi2W0.5Mo0.5O6 solid solution nanotubes under visible light. RSC Adv. 10, 8821–8824. 10.1039/d0ra00672f PMC9049998 35496522

[B49] Wang Y. Shen S. H. Shen S. (2020b). Progress and prospects of non-metal doped graphitic carbon nitride for improved photocatalytic performances. Acta Phys. Chim. Sin. 36, 1905080. 10.3866/pku.whxb201905080

[B42] Wu J. Q. Hua W. M. Yue Y. H. Gao Z. (2020). Swelling characteristics of g-C_3_N_4_ as base catalyst in liquid-phase reaction. Acta Phys. Chim. Sin. 36, 1904066. 10.3866/pku.whxb201912023

[B14] Yan S. C. Li Z. S. Zou Z. G. (2010). Photodegradation of rhodamine B and methyl orange over boron-doped g-C_3_N_4_ under visible light irradiation. Langmuir 26, 3894–3901. 10.1021/la904023j 20175583

[B25] Yang Y. Chen J. Mao Z. An N. Wang D. Fahlman B. D. (2017). Ultrathin g-C_3_N_4_ nanosheets with an extended visible-light-responsive range for significant enhancement of photocatalysis. RSC Adv. 7, 2333–2341. 10.1039/c6ra26172h

[B2] Yu Z. Li F. Yang Q. Shi H. Chen Q. Xu M. (2017). Nature-mimic method to fabricate polydopamine/graphitic carbon nitride for enhancing photocatalytic degradation performance. ACS Sustainable Chem. Eng. 5, 7840–7850. 10.1021/acssuschemeng.7b01313

[B1] Yu C. He H. Liu X. Zeng J. Liu Z. (2019). Novel SiO_2_ nanoparticle-decorated BiOCl nanosheets exhibiting high photocatalytic performances for the removal of organic pollutants. Chinese J. Catal. 40, 1212–1221. 10.1016/s1872-2067(19)63359-0

[B8] Zhang D. Tan G. Wang M. Li B. Dang M. Ren H. (2020). The modulation of g-C_3_N_4_ energy band structure by excitons capture and dissociation. Mater. Res. Bull. 122, 110685. 10.1016/j.materresbull.2019.110685

[B37] Zhang P. Li Y. Zhang Y. Hou R. Zhang X. Xue C. (2020a). Photogenerated electron transfer process in heterojunctions: in situ irradiation XPS. Small Methods 4, 2000214. 10.1002/smtd.202000214

[B33] Zhang P. Tong Y. Liu Y. Vequizo J. J. M. Sun H. Yang C. (2020b). Heteroatom dopants promote two-electron O_2_ reduction for photocatalytic production of H_2_ O_2_ on polymeric carbon nitride. Angew. Chem. Int. Ed. Engl. 59, 16209–16217. 10.1002/anie.202006747 32430972

[B48] Zhang P. Zhang S. Wan D. Zhang P. Zhang Z. Shao G. (2020c). Multilevel polarization-fields enhanced capture and photocatalytic conversion of particulate matter over flexible schottky-junction nanofiber membranes. J. Hazard. Mater. 395, 122639. 10.1016/j.jhazmat.2020.122639 32305717

[B41] Zhang S. Zhang P. Hou R. Li B. Zhang Y. Liu K. (2020). In situ sulfur-doped graphene nanofiber network as efficient metal-free electrocatalyst for polysulfides redox reactions in lithium-sulfur batteries. J. Energy Chem. 47, 281–290. 10.1016/j.jechem.2020.01.033

[B39] Zhang Y. Zhang P. Li B. Zhang S. Liu K. Hou R. (2020). Vertically aligned graphene nanosheets on multi-yolk/shell structured TiC@C nanofibers for stable Li-S batteries. Energy Storage Mater. 27, 159–168. 10.1016/j.ensm.2020.01.029

[B24] Zhao S. Zhang Y. Zhou Y. Wang Y. Qiu K. Zhang C. (2018). Facile one-step synthesis of hollow mesoporous g-C3N4 spheres with ultrathin nanosheets for photoredox water splitting. Carbon 126, 247–256. 10.1016/j.carbon.2017.10.033

[B30] Zheng D. Pang C. Liu Y. Wang X. (2015). Shell-engineering of hollow g-C3N4 nanospheres via copolymerization for photocatalytic hydrogen evolution. Chem. Commun. 51, 9706–9709. 10.1039/c5cc03143e 25980518

